# Efficacy and Safety of Various Treatments for Proliferative Diabetic Retinopathy: A Systematic Review and Network Meta-Analysis

**DOI:** 10.3389/fphar.2021.709501

**Published:** 2021-11-09

**Authors:** Bo Zhang, Zhulin Zhou, Bingjie Zhang, Dan Wang

**Affiliations:** ^1^ Department of Neurosurgery, First Hospital of Jilin University, Changchun, Jilin, China; ^2^ Department of Neurobiology, Care Sciences & Society, Division of Neurodegerneration, Karolinska Institutett, Karolinska University Hospital Huddinge, Stockholm, Sweden; ^3^ Department of Ophthalmology, First Hospital of Jilin University, Changchun, Jilin, China

**Keywords:** proliferative diabetic retinopathy, systematic review, network meta-analysis, laser photocoagulation, anti-VEGF (vascular endothelial growth factor)

## Abstract

Diabetic retinopathy is the main cause of visual impairment and blindness. The proliferative diabetic retinopathy at the severe stage of diabetic retinopathy is more harmful to vision and even leads to total blindness. To evaluate the visual acuity, central retinal thickness, and adverse reactions of various treatments for proliferative diabetic retinopathy through a systematic network meta-analysis. The relevant research published in English or Chinese from January 1, 2011, to February 1, 2021, was systematically searched by using PubMed, science network, EMBASE, MEDLINE, Cochrane Library, China National Knowledge Infrastructure, Wanfang, and other electronic databases. A total of 15 studies were selected, including 3,222 eyes of PDR patients. Our results show that in terms of visual score improvement, ranibizumab alone (69.90%) and laser + ranibizumab (67.90%) are the best. However, if the groups were grouped again according to the dose and times of ranibizumab injection, the results showed that 0.5 mg ranibizumab injection per month (58.0%) had the best effect on vision improvement. For the change of central retinal thickness, the thickness decreased the most after the laser combined with ranibizumab (96.5%). After the same subgroup analysis, the results were further refined into the best effect of laser combined with 0.3 mg ranibizumab per quarter (72.7%). In addition, our analysis of complications also showed that the overall incidence of adverse reactions of PRP (11.1 ± 12.4, %) was greater than that of ranibizumab (10.6 ± 13.0, %). However, more high-quality randomized controlled trials with longer follow-up using standard methods are still needed to verify the correlation.

## Introduction

With the increasing prevalence of diabetes worldwide, the incidence rate of diabetic retinopathy (DR) is also increasing. It is reported that the annual incidence rate of DR is about 2.2–12.7% (Sabanayagam et al., 2019). DR is a common and specific microvascular complication in diabetes mellitus, and it is the main reason for preventable blindness in adults. About 10% of diabetics are affected by DR (including proliferative DR (PDR)) or diabetic macular edema (DME) with vision threat (Ebneter and Zinkernagel, 2016). PDR is a kind of vision-threatening complication of DR, which is characterized by abnormal new vessels in the retina, optic nerve head, or anterior segment (Moutray et al., 2018).

Treatment options for PDR include laser photocoagulation (all kinds of lasers except pan-retinal photocoagulation (PRP), and this paper mainly refers to focal/grid laser photocoagulation), PRP, anti-vascular endothelial growth factor (anti-VEGF) therapy, and vitrectomy. Early, laser photocoagulation is the mainstay of treatment for PDR but is gradually being superseded for DME. Timely laser treatment has a good protective effect on the vision of patients with PDR, but the ability to reverse the decline of vision is poor. PRP has gradually become the main treatment for PDR, although laser treatment may still be important in treating early cases (Heng et al., 2013). However, although PRP has been the standard treatment for decades, some recent clinical trials show that anti-VEGF therapy is a reasonable choice for PRP in the treatment of PDR (Sun et al., 2019). Vitreous VEGF concentrations are elevated in patients with PDR (Aiello et al., 1994). The new treatment, intravitreal injection of anti-vascular endothelial growth factor drugs, is less destructive to the retina than other treatments and may be useful for patients with adverse reactions to conventional treatment. Currently, preoperative anti-VEGF therapy has been widely used as an adjunct for PDR surgery (Chen et al., 2020). Vitrectomy is typical retention of non-clear vitreous hemorrhage or traction retinal detachment threat of macular involvement. It is sometimes necessary for advanced retinopathy.

The decision of which treatment to use will vary according to the specific situation of each patient, and the treatment decision should consider the relative advantages of each treatment. Thus, a high-quality review, comparing various treatment methods, was necessary. The existing research mainly focuses on DME, with less review on the treatment of PDR and treatment. This systematic review was designed to comprehensively examine the changes in vision and safety of various treatments in patients with PDR to find out the advantages and disadvantages of various treatment methods and provide a reference for the selection of treatment methods for different subtypes of the PDR population.

## Materials and Methods

### Search Strategy

The paper was designed to answer the following focused question: “Effects of various treatments (including dosage and administration frequency) on visual acuity and adverse reactions in patients with PDR”. Using PubMed, EMBASE, Medicine, Cochrane Library, China National Knowledge Infrastructure (CNKI), Wanfang, the relevant research published in English or Chinese from January 1, 2011, to February 1, 2021, was searched systematically. The key search words are as follows diabetic retinopathy or diabetic macular edema or diabetic eye disease, proliferative or proliferation. In addition, references cited in each included study, as well as relevant systematic reviews and meta-analyses, were manually searched to identify potentially relevant studies.

### Eligibility Criteria

The language was limited to English and Chinese, and only randomized controlled trials (RCTs) were included. The type of article is the comparison of treatment methods of PDR patients between at least two groups, and a follow-up period of at least 1 year. The criteria for the included population in these studies were at least 18 years old, with type 1 or type 2 diabetes, at least one eye with PDR, and no previous PRP. According to the international clinical grading standards for DR and DME, the five-grade disease severity grading of diabetic retinopathy includes three low-risk stages, the fourth stage of severe no proliferative retinopathy, and the fifth stage of proliferative retinopathy. DME is divided into obvious presence or obvious absence (Wu et al., 2013). Therefore, the literature which only described diabetic macular edema but did not describe proliferative diabetic retinopathy in detail was excluded. The number of eyes treated, the comparison of various indicators before and after treatment, the dose and frequency of each treatment, and adverse reactions must be included in the study. And if two or more treatments were included in one RCT, the study must provide the number of patients assigned to each treatment and the effect of the treatment.

Other exclusion criteria are: 1) review articles, letters, meeting abstracts, personal opinions, and book chapters; 2) articles without complete data; 3) articles that cannot be converted to a unified data format; 4) articles that do not display the original results; 5) complete articles that cannot be fully accessed; 6) articles that do not know whether it is proliferative. If multiple publications based on the same cohort are identified, the report with the largest number of patients is used. When the number of patients was the same, the latest published articles were selected.

### Search and Selection Strategy

All searches were conducted in February 2021. PubMed, EMBASE, Medicine, Cochrane Library, CNKI, Wanfang, and other literature databases were used. At the end of the search phrase, the reference list of each selected article is manually screened to find the relevant research that may be missed in the process of database search. The articles in the study were selected by two authors (Bo Zhang. and Dan Wang.). According to the qualification criteria, the title and abstract are independently evaluated, and then independently searched and the full text is evaluated. The differences between the commentators were resolved by the consensus of another author (Bingjie Zhang), who was finally selected for the paper.

### Data Extraction Process and Data Items

Two authors (Bo Zhang. and Bingjie Zhang.) collected the data. The third author, Dan Wang, cross-checked all the information. All studies recorded the following descriptive features: author, year, inclusion and exclusion criteria, number of eyes, average age and gender of each group, follow-up period, administration mode, baseline data, average/standard, method, results, and conclusions. All data were converted to mean and standard deviation. If the original study reported the average and standard deviation of the result variable directly, the reported values were used directly. If the original study did not directly report the mean value and standard deviation of the result variable but reported the average value and standard deviation of the result variable before and after the intervention, the average value and standard deviation of the change of the result variable before and after intervention were calculated according to the method in article 16.1.3.2 of Cochrane Handbook 5.0.2.

### Risk of Bias in Individual Studies

The methodological quality of the study was assessed using the Cochrane bias risk assessment tool. The two authors (Bo Zhang. and Zhulin Zhou.) independently extracted data and evaluated the methodological quality of the trial. The results were compared and any differences were discussed by the third author (Dan Wang.) if necessary. The risk of bias includes seven parts: 1) random sequence generation, 2) allocation hiding, 3) blind approach for participants and personnel, 4) blind method for results evaluation, 5) incomplete result data, 6) selective reporting, and 7) other sources of bias. We divided the test into three risk levels based on the number of components that may have high bias risk: high risk (5 or more), medium risk (3 or 4), and low risk (2 or less). The quality of the study ranges from high to low, excluding highly biased literature.

### Statistical Analyses

Stata software 15.0 (StataCorp, College Station, TX, United States) was used for all statistical analyses. Our study outcomes included a variety of efficacy indicators and the number of adverse reactions. In order to evaluate the therapeutic effect of different therapeutic indexes on different degrees of diabetic retinopathy, we first used meta-analysis with Stata command. The heterogeneity between studies was evaluated by Q-test and further quantified by the *I* (Cheung et al., 2010) index. If there was significant heterogeneity (*p* < 0.05 or *I* ^*2*^ > 50%), a random effect model was used to collect ORs, the results were expressed as SMD and relative 95% CI; otherwise, a fixed-effect model was used. If no event occurred in at least one arm, the number of cases and non-cases increased by 0.5 per arm. Dichotomous data, such as adverse events, were analyzed by random effect model and the risk difference (RD) of 95% CI was used to summarize the results of each treatment group.

Secondly, we used the Stata command “mvmeta” to analyze the random effect network in the framework of frequency. In order to verify the consistency hypothesis in the network, the interaction model based on the design of treatment was adopted. Inconsistencies were assessed by the loop-specific approach and the side-splitting model. The surface under the cumulative ranking curve (SUCRA) and the ranking probability distribution were used to estimate and rank the best treatment regimens. A network graph was generated for each network meta-analysis, and the publication bias was evaluated visually by drawing a funnel chart for comparison and correction. Two formal tests (Begg rank correlation test and Egger regression asymmetry test) were used to test the asymmetry of the funnel plot. The risk of comparison-specific bias was estimated for each available direct comparison treatment indicator. Bilateral *p* < 0.05, with statistical significance.

## Results

### Study Selection and Study Characteristics

**FIGURE 1:**
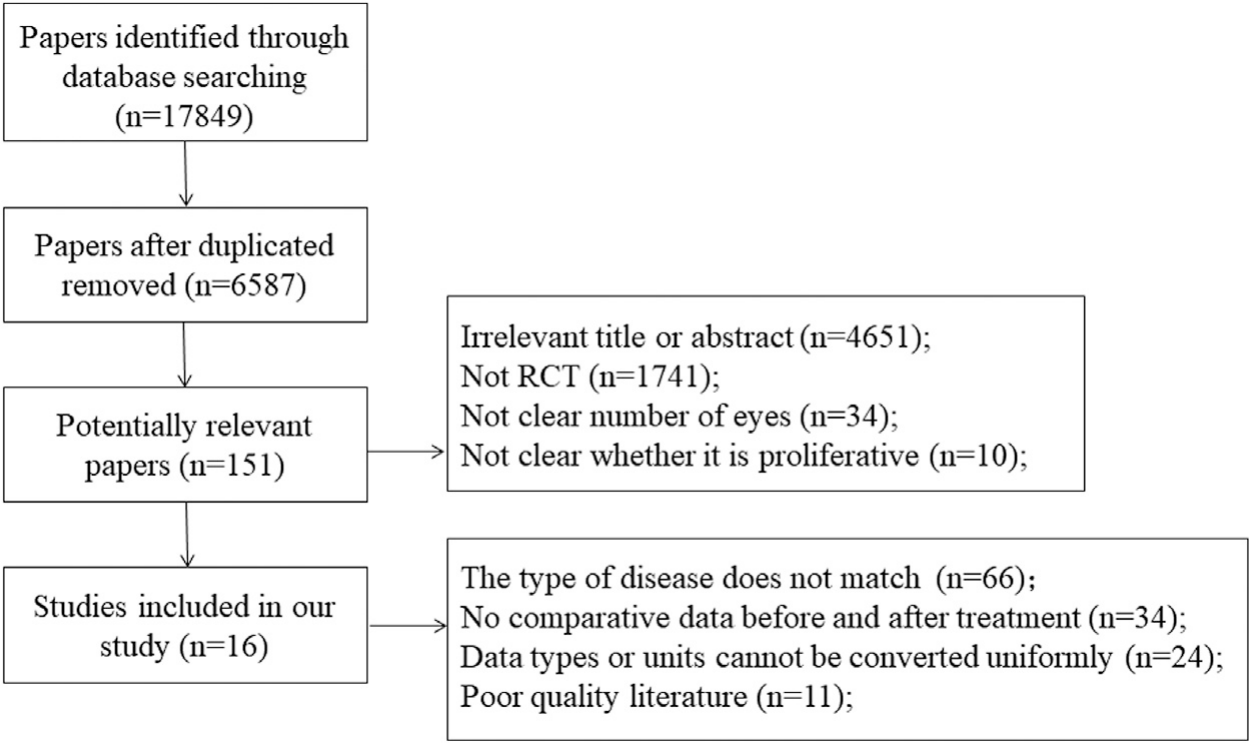
Flow chart of literature search and study selection.

In the first phase, 17,849 studies were selected from six electronic databases. After deleted repeated researchers, 6,587 citations were retained. Subsequently, 6,436 citations were excluded due to comprehensively evaluated research relevance, therefore, 151 studies were included in the second stage. In the end, taking some factors into account, such as article quality and so on, 15 studies met the inclusion criteria. Figure 1 shows a flow chart detailing the identification, inclusion, and exclusion process of the study.

The studies selected to comprise the present review were published between 2011 and 2019 and were all written in the English language. They were conducted in five different countries: United States (Mitchell et al., 2011; Beaulieu et al., 2016; Gross et al., 2016; Bressler et al., 2017a; Bressler et al., 2017b; Gross et al., 2017; Bressler et al., 2018; Gross et al., 2018; Payne et al., 2019; Wykoff et al., 2019) Portugal (Figueira et al., 2018), United Kingdom (Sivaprasad et al., 2017), Denmark (Scott et al., 2014), and Germany (Lang et al., 2018). All selected articles were RCT-based studies. 15 RCTs included nine interventions: control group, PRP, laser, and seven drug treatments, including four drugs (Ranibizumab, Aflibercept, Bevacizumab, and Doxycycline Monohydrate). There were two groups of administration frequency: monthly administration and quarterly (3–4 months) administration. Three studies were three-arm trials, and the remaining 12 studies were two-arm trials. Other descriptive information for each study was listed in Supplementary Appendix 1. Table 1 shows the characteristics of the 15 RCTs that included 3,222 eyes. Study sample size ranged from 21 to 394 (median, 202 eyes). In all RCTs, the mean age was from 48 to 63.5 years. The median of the follow-up period was 2 years (range, 1–5 years). Supplementary Appendix 2 summarizes the treatment options for each study, including dose and frequency. Supplementary Appendix 3 summarizes the characteristics of included RCTs related to study quality, and the bias risk assessment based on these characteristics, using the bias risk assessment tool of Cochrane Collaboration. As for the risk of bias, four trials (18, 21, 23, 23) were judged as low risk of bias, and the other 11 trials were judged as medium risk of bias. Supplementary Appendix 4–6 summarizes the main adverse reactions in all studies.

**TABLE 1:** Network meta-analysis of vision score change.

Ranibizumab	-	-	-	-
−0.00 (−0.61,0.61)	laser + ranibizumab	-	-	-
0.15 (−0.35,0.65)	0.15 (−0.64,0.94)	PRP	-	-
0.16 (−0.83,1.15)	0.16 (−1.00,1.33)	0.01 (−0.84,0.87)	aflibercept	-
0.74 (−0.06,1.55)	0.74 (−0.06,1.55)	0.59 (−0.35,1.54)	0.58 (−0.70,1.86)	laser
69.90%	67.90%	51.40%	51.10%	9.70%

Treatment reports were sorted according to the degree of vision improvement. Comparisons should be read from left to right. The estimate is located at the intersection of the column-defining treatment and the row defining treatment. Values in parenthesis indicate the 95% CI. Values in the last row indicate the SUCRA value of the corresponding column.

### Vision Changes

**FIGURE 2:**
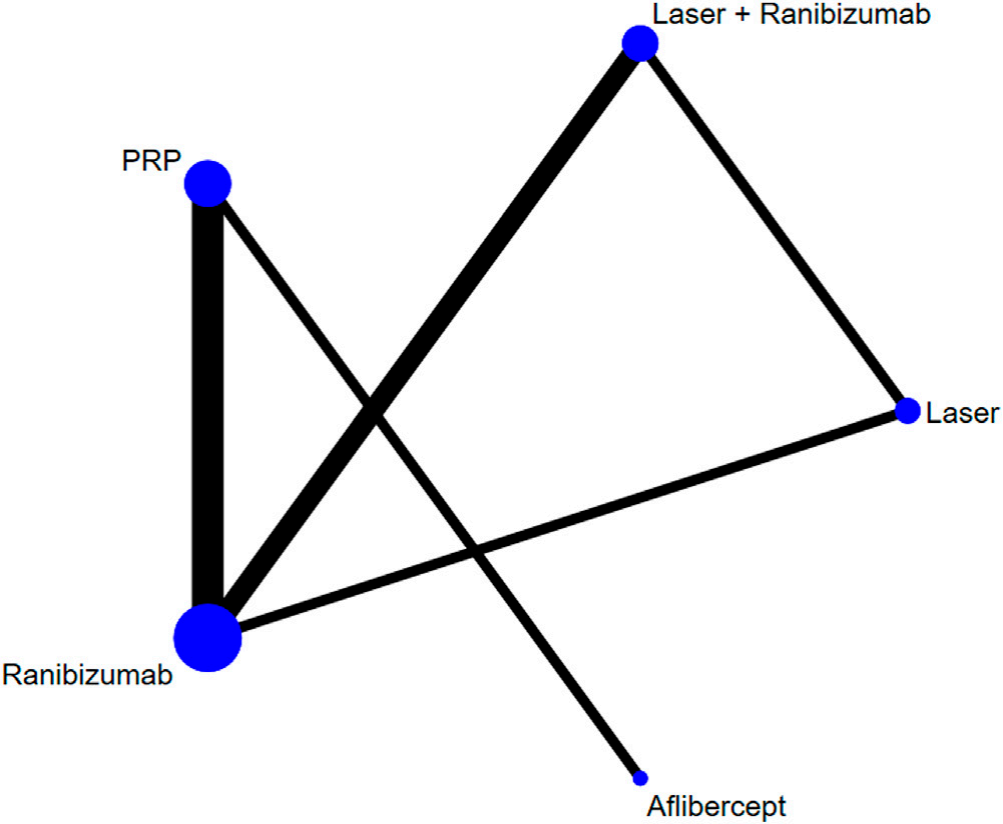
Network of comparisons of various treatments for vision score change. Seven studies involving 1,136 eyes were included. Five interventions were included to form a closed loop. Each circle indicates a treatment node. The size of the nodes is proportional to the number of trials evaluating each treatment. Lines connecting two nodes represent direct comparisons between two treatments. The thicker the number of lines between nodes, the more research.

**FIGURE 3:**
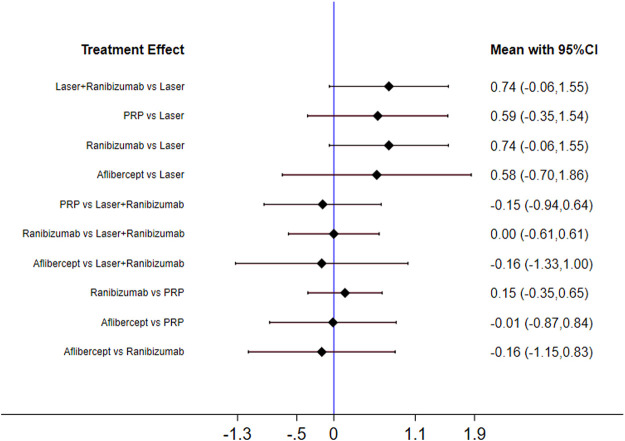
Forest plots for head-to-head comparisons of SMD of vision score change. Horizontal lines indicate the range of 95% CI.

Figure 2 shows the control group with direct evidence and describes the trial network used in the meta-analysis of changes in visual acuity scores with various treatments (7 trials, 1,136 eyes). Of the 45 pairs available for comparison, 10 had direct evidence. Among the 13 pairs of subjects, the multi-arm (more than 3) trial involved the comparison of 10 pairs of subjects. The results of the paired meta-analysis are shown in Figure 3. For vision improvement, laser combined with ranibizumab (67.90%) and ranibizumab alone (69.90%) was the best, but ranibizumab alone was slightly better. The effect of PRP (51.40%) and aflibercept (51.10%) was the second. Laser alone (9.70%) has the worst effect (Table 1). When compared with laser alone, whether ranibizumab and laser were combined or not has no difference in the improvement of visual score [STD Mean Difference (SMD) = 0.74; 95% CI (-0.06, 1.55)]. Similarly, when compared with aflibercept alone, whether ranibizumab and laser were combined or not has no difference in the improvement of visual score [aflibercept vs laser + ranibizumab: SMD = −0.16; 95% CI (−1.33, 1.00); aflibercept vs ranibizumab: SMD = −0.16; 95% CI (−1.15, 0.83)]. However, aflibercept shows better improvement than laser [aflibercept vs laser: SMD = 0.58; 95% CI (−0.70, 1.86)] (Figure 3). Funnel plots (Supplementary Appendix 10), Begg’s tests (Supplementary Appendix 11), and Egger’s tests (Supplementary Appendix 12, *p* = 0.411) demonstrate the lack of publication bias for any of the analyses.

**FIGURE 4:**
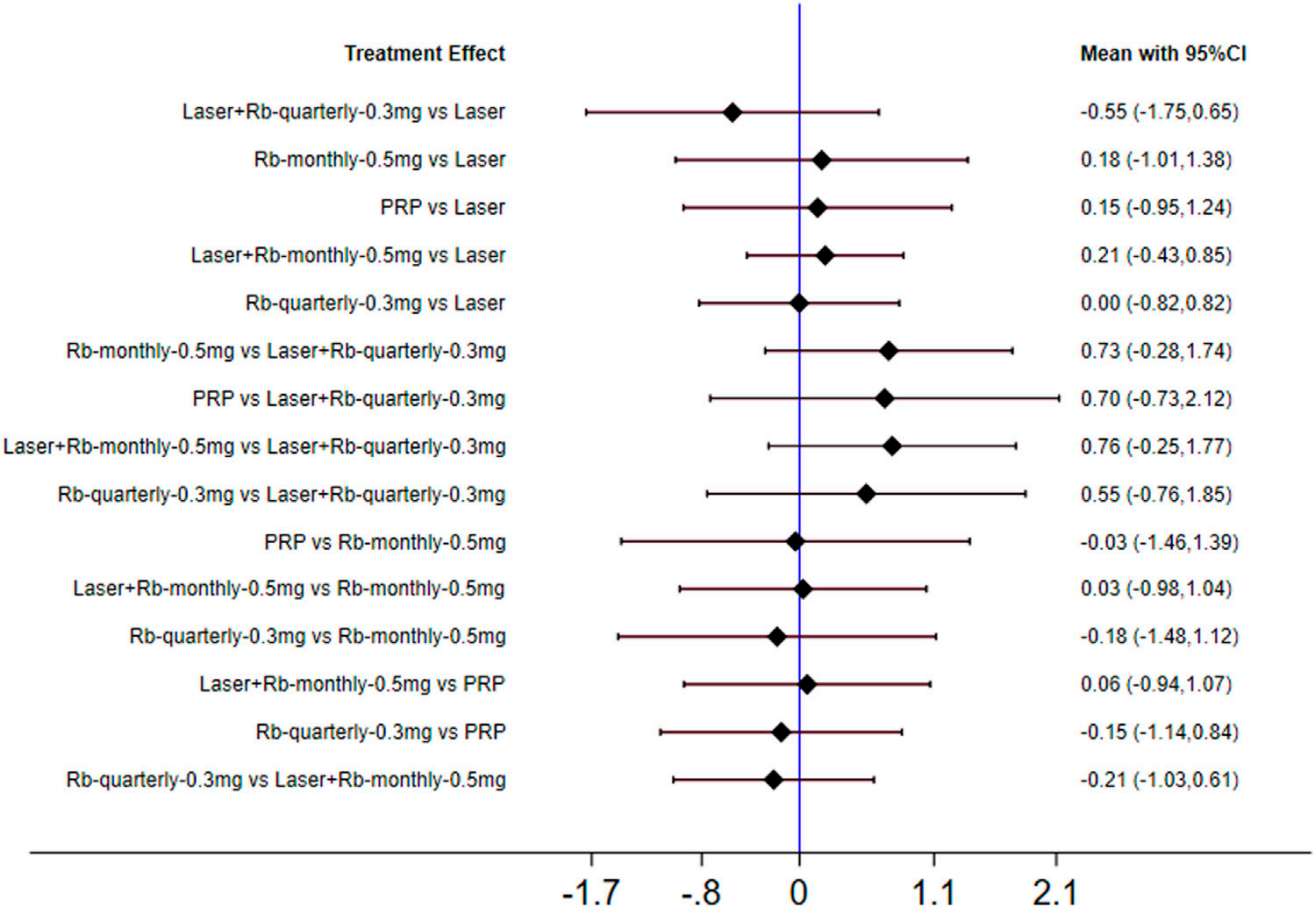
Forest plots for subgroup analysis of vision score change. Horizontal lines indicate the range of 95% CI. Abbreviation: Ranibizumab = Rb.

This phenomenon may be related to the injection frequency and dose of ranibizumab, so we conducted a subgroup analysis on the injection frequency and dose of ranibizumab. This phenomenon may be related to the injection frequency and dose of ranibizumab. Therefore, we conducted subgroup analysis on the injection frequency and dose of ranibizumab. The results of forest plots are shown in Figure 4. The effect of each treatment on the improvement of visual acuity score was similar (ranibizumab 0.5 mg/month: 58.0%, laser: 54.5%, laser + ranibizumab 0.5 mg/month: 54.0%, ranibizumab 0.5 mg/quarter: 53.7%, PRP: 49.1%, aflibercept 2 mg/quarter: 47.3%.). When comparing the efficacy of laser combined with ranibizumab and other treatment methods, the injection dose and injection frequency of ranibizumab have a greater impact on the improvement of visual acuity. Among them, laser + ranibizumab 0.5 mg/month was better than laser + ranibizumab 0.3 mg/quarter, showing the vision improvement with the largest gap [laser + ranibizumab 0.5 mg/month vs laser + ranibizumab 0.3 mg/quarter: SMD = 0.76; 95% CI (−0.25, 1.77)], but the effect of laser + ranibizumab 0.3 mg per quarter was the worst than laser [laser + ranibizumab 0.5 mg/quarter vs laser: SMD = −0.55; 95% CI (−1.73, 0.65)] (Figure 4). Other results are shown in Supplementary Appendices 7–18.

### Central Retinal Thickness

**FIGURE 5:**
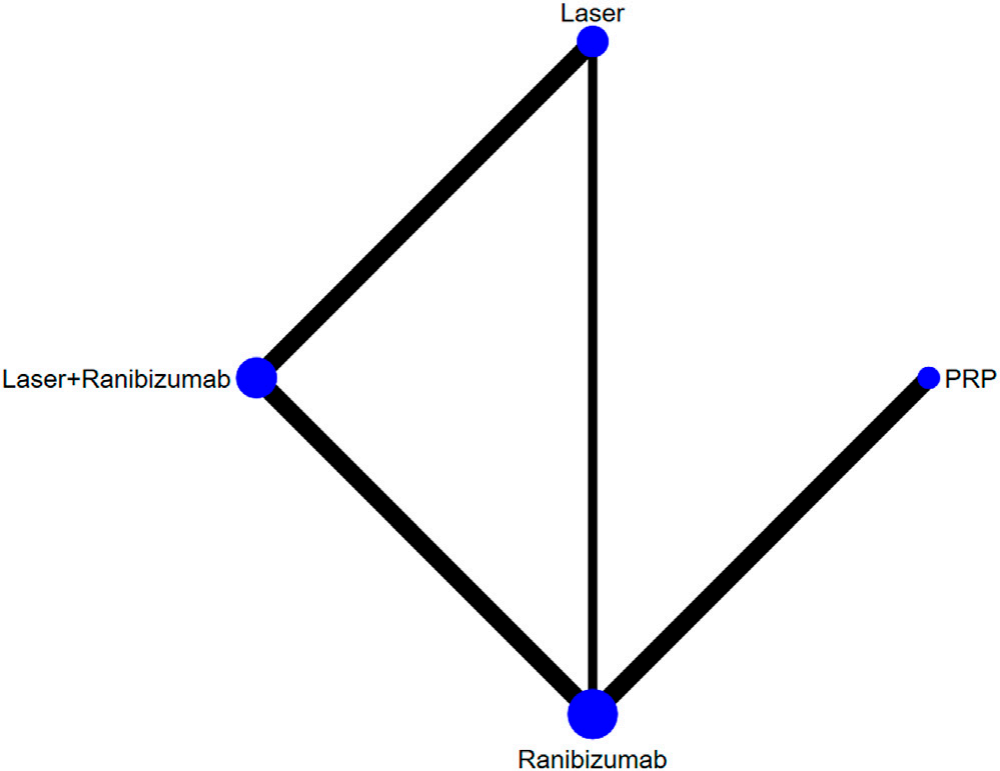
Network of comparisons of various treatments for central retinal thickness. Five studies involving 1,411 eyes were included. Four interventions were included to form a closed loop. Each circle indicates a treatment node. The size of the nodes is proportional to the number of trials evaluating each treatment. Lines connecting two nodes represent direct comparisons between two treatments. The thicker the number of lines between nodes, the more research.

**FIGURE 6:**
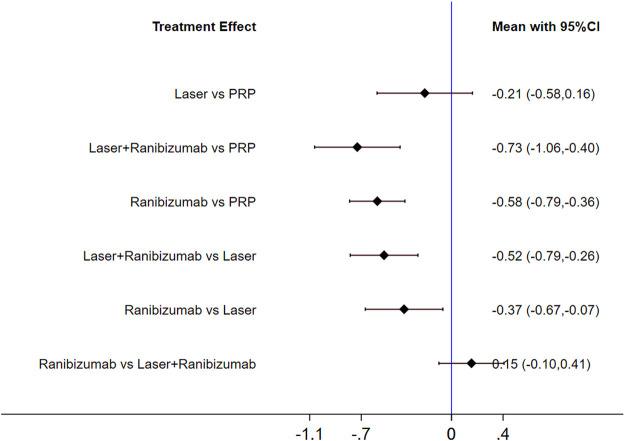
Forest plots for head-to-head comparisons of SMD of central retinal thickness. Horizontal lines indicate the range of 95% CI.

Figure 5 shows the control group with direct evidence and describes the trial network used in the meta-analysis of changes in central retinal thickness with various treatments (5 trials, 1,411 eyes). The results of the paired meta-analysis are shown in Figure 6. For central retinal thickness, laser combined with ranibizumab (96.5%) was the best (Table 2). The effect of ranibizumab (69.9%) and laser (29.4%) was the second. PRP alone (4.2%) had the worst effect (Supplementary Appendices 7–9). Compared with PRP and other therapies, laser combined with ranibizumab had the greatest effect on reducing the central retinal thickness [laser + ranibizumab vs PRP: SMD = −0.73; 95% CI (−1.06, −0.40)]. For ranibizumab, the difference of reduced central retinal thickness with or without laser was the smallest [ranibizumab vs laser + ranibizumab: SMD = 0.15; 95% CI (−0.10, 0.41)] (Figure 6). Funnel plots (Supplementary Appendix 22), Begg’s tests (Supplementary Appendix 23), and Egger’s tests (Supplementary Appendix 24, *p* = 0.222) demonstrate the lack of publication bias for any of the analyses.

**TABLE 2:** Network meta-analysis of central retinal thickness.

laser + ranibizumab	-	-	-
−0.15 (−0.41,0.10)	Ranibizumab	-	-
−0.52 (−0.79,−0.26)	−0.37 (−0.67,−0.07)	laser	-
−0.73 (−1.06,−0.40)	−0.58 (−0.79,−0.36)	−0.21 (−0.58,0.16)	PRP
96.5%	69.9%	29.4%	4.2%

Treatment reports were sorted according to the central retinal thickness. Comparisons should be read from left to right. The estimate is located at the intersection of the column-defining treatment and the row-defining treatment. Values in parenthesis indicate the 95% CI. Values in the last row indicate the SUCRA value of the corresponding column.

**FIGURE 7:**
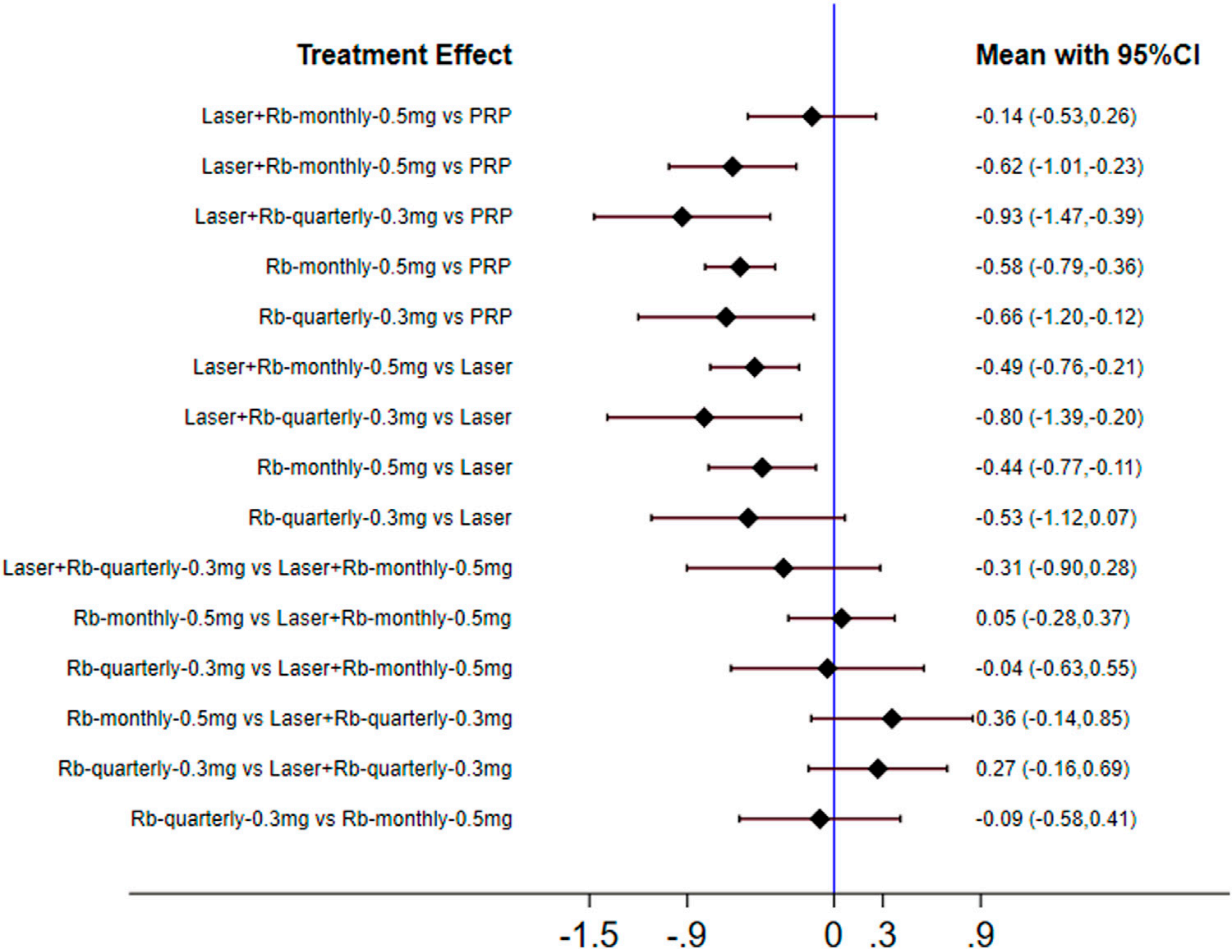
Forest plots for subgroup analysis of central retinal thickness. Horizontal lines indicate the range of 95% CI. Abbreviation: Ranibizumab = Rb.

Similarly, we performed a subgroup analysis of the injection frequency and dose of ranibizumab. The results of forest plots are shown in Figure 7. The efficacy of each subgroup in reducing central retinal thickness was as follows: laser + ranibizumab 0.3 mg/quarter (72.7%), ranibizumab 0.3 mg/quarter (45.7%), laser + ranibizumab 0.5 mg/month (44.3%), ranibizumab 0.5 mg/month (36.8%), laser (26.2%), and PRP (20.1%). When comparing the efficacy of laser combined with ranibizumab and other treatment methods, the injection dose and injection frequency of ranibizumab have a greater impact on the improvement of visual acuity. Compared with PRP and other therapies, laser combined with quarterly injection of ranibizumab 0.3 mg had the greatest effect on reducing central retinal thickness [laser + ranibizumab 0.3 mg/quarter vs PRP: SMD = −0.93; 95% CI (- 1.47, - 0.39)]. The effect of ranibizumab 0.5 mg per month was the worst compared with laser combined with ranibizumab 0.3 mg per quarter. [ranibizumab 0.5 mg/month vs laser + ranibizumab 0.3 mg/quarter: SMD = 0.36; 95% confidence interval (− 0.14,0.85)] (Figure 7). Other results are shown in Supplementary Appendices 19–29.

## Discussion

DR is the main cause of vision loss in adults. Visual impairment caused by diabetic retinopathy has a serious negative influence on the quality of life of patients (Hendrick et al., 2015). The severe stage of DR can be caused by abnormal growth of retinal vessels and DME. At present, there are many treatments for DR, but there are different opinions on which treatment to take. Studies have shown that anti-VEGF therapy can be used for DME combined with vision loss, and laser photocoagulation can prevent severe vision loss caused by PDR (Wong et al., 2016). However, there is still a lack of high-quality reviews to draw a clear conclusion. In this study, we conducted a comprehensive review of the RCT of various treatment methods for patients with PDR to draw a conclusion.

Firstly, in terms of visual score improvement, ranibizumab alone (69.90%) and laser + ranibizumab (67.90%) were the best. However, if the groups were grouped again according to the dose and times of ranibizumab injection, the results showed that 0.5 mg ranibizumab injection per month (58.0%) had the best effect on vision improvement. The second was laser therapy alone (54.5%). For the change of central retinal thickness, the thickness decreased the most after the laser combined with ranibizumab (96.5%). After the same subgroup analysis, the results were further refined into the best effect of laser combined with 0.3 mg ranibizumab per quarter (72.7%). In addition, most studies tend to believe that the side effects of laser therapy were higher than those of drug therapy (Lang et al., 2018; Barth and Helbig, 2021). Our analysis of complications also showed that the overall incidence of adverse reactions of PRP (11.1 ± 12.4, %) was greater than that of ranibizumab (10.6 ± 13.0, %).

Therefore, considering the efficacy, adverse reactions, and economy, our meta-analysis suggests that we should choose the appropriate treatment scheme according to the focus of improvement needed by different disease populations. However, our research still has many limitations. Even if we do not limit the analysis to location or evaluation methods in the database search process, we only include English and Chinese studies. In addition, considering that various therapies have been greatly updated and improved in recent decades, to reduce bias, we only considered RCT in the last decade. Similarly, due to the limitations of the research literature, we cannot further limit and classify various treatment methods, such as scanning range or wavelength. And after grouping, the number of studies in each group is further reduced and the reliability of the conclusions is further reduced. In addition, diabetes duration, blood pressure control, and blood glucose control may be confounded factors, which were not considered in this analysis because of limited data collection.

## Data Availability

The original contributions presented in the study are included in the article/Supplementary Material, further inquiries can be directed to the corresponding author.
