# Supplementary material for: Efficacy and Safety of Various Treatments for Proliferative Diabetic Retinopathy: A Systematic Review and Network Meta-Analysis
Source: Front Pharmacol. 2021 Nov 9;12:709501. doi: 10.3389/fphar.2021.709501 (PMC8630659; doi:10.3389/fphar.2021.709501)
Supplement: Supplementary file 1 [file DataSheet1.DOCX]

**Appendix 1. Summarized descriptive characteristics of included studies**

| **author** | **year** | **Country** | **Follow up time** | **Age-mean** | **Team 1** | **Team 2** | **Eye number** | | **Women** | | **Men** | | **Type I** | | **Type II** | |
| --- | --- | --- | --- | --- | --- | --- | --- | --- | --- | --- | --- | --- | --- | --- | --- | --- |
|  |  |  |  |  |  |  | **team 1** | **team 2** | **team 1** | **team 2** | **team 1** | **team 2** | **team 1** | **team 2** | **team1** | **team2** |
| Bressler SB | 2018 | U.S. | 2 years | - | Ranibizumab | PRP | 147 | 155 | 71 | 77 | 89 | 91 | 38 | 30 | 117 | 131 |
| Figueira J | 2018 | Portugal | 1 year | 54.8 | Ranibizumab + PRP | PRP | 41 | 46 | 13 | 19 | 28 | 27 | - | - | - | - |
| Gross JG | 2018 | U.S. | 5 years | 52 | Ranibizumab | PRP | 191 | 203 | 135 | | 170 | | 30 | 24 | 83 | 93 |
| Sun JK | 2019 | U.S. | 2 years | - | Ranibizumab | PRP | 191 | 203 | - | - | - | - | - | - | - | - |
| Gross JG | 2015 | U.S. | 2 years | 52 | Ranibizumab | PRP | 191 | 203 | 83 | 92 | 108 | 111 | 43 | 41 | 140 | 155 |
| Gross JG | 2017 | U.S. | 5 years | - | Ranibizumab | PRP | 102 | 114 | - | - | - | - | - | - | - | - |
| Susan B. Bressler | 2017 | U.S. | 2 years | 52 | Ranibizumab | PRP | 191 | 203 | 83 | 92 | 108 | 111 | 28 | 44 | 32 | 36 |
| Beaulieu WT | 2016 | U.S. | 2 years | - | Ranibizumab | PRP | 102 | 114 | 43 | 52 | 59 | 62 | 21 | 19 | 76 | 91 |
| Susan B. Bressler | 2017 | U.S. | 2 years | 61 | Aflibercept | Bevacizumab-1 | 47 | 59 | - | - | - | - | 8 | 35 | 7 | 47 |
|  |  |  |  |  | Bevacizumab-1 | Ranibizumab-2 | 59 | 49 | - | - | - | - | 7 | 47 | 7 | 33 |
| Sivaprasad S | 2017 | UK | 52weeks | 51.15 | Aflibercept | PRP | 116 | 33 | 116 | 83 | 44 | 72 | 51 | 65 | 54 | 62 |
| Wykoff CC NMZB | 2019 | U.S. | 1 year | 48 | Aflibercept | Aflibercept | 20 | 11 | 20 | 9 | 8 | 12 | - | - | - | - |
| Scott IU | 2014 | Denmark | 2 years | 54.7 | CON | Doxycycline Monohydrate | 15 | 6 | 15 | 9 | 6 | 9 | - | - | - | - |
| Mitchell P | 2011 | U.S. | 1 year | 63.45 | Ranibizumab-1 | Ranibizumab-2 | 116 | 43 | 118 | 73 | 48 | 70 | 13 | 103 | 15 | 102 |
|  |  |  |  | 63.19 | Ranibizumab-1 | Laser | 116 | 43 | 111 | 73 | 53 | 58 | 15 | 102 | 13 | 97 |
| Payne JF | 2019 | U.S. | 2 years | - | Ranibizumab-1 | Ranibizumab-2 | 30 | 60 | - | - | - | - | - | - | - | - |
|  |  |  |  |  | Ranibizumab-1 | Laser +Ranibizumab | 30 | 60 | - | - | - | - | - | - | - | - |
| Lang GE | 2018 | Germany | 12 months | 63.5 | Laser +Ranibizumab | Laser | 85 | 32 | 43 | 53 | 16 | 27 | - | - | - | - |

**Appendix 2. Methods of Administration for included patients**

| **author** | **Methods of Administration for team 1** | **Methods of Administration for team 2** | **Average treatment times for team 1** | **Average treatment times for team 2** |
| --- | --- | --- | --- | --- |
| Bressler SB | Ranibizumab-monthly-0.5 mg | PRP | - | - |
| Figueira J | Ranibizumab-quarterly-0.5 mg + PRP | PRP | - | - |
| Gross JG | Ranibizumab-monthly-0.5 mg | PRP | 19.2±10.9 | 5.4±7.9 |
| Sun JK | Ranibizumab-monthly-0.5 mg | PRP | 10.0±24.7 | 10.0±25.4 |
| Gross JG | Ranibizumab-monthly-0.5 mg | PRP | - | - |
| Sivaprasad S | Aflibercept-quarterly- 2 mg | PRP | - | - |
| Wykoff CC NMZB | Aflibercept-monthly-2 mg | Aflibercept-quarterly-2 mg | - | - |
| Scott IU | CON | Doxycycline Monohydrate- daily-50 mg | - | - |
| Mitchell P | Ranibizumab-monthly- 0.5 mg | Ranibizumab-monthly-0.5 mg + Laser | 7±2.81 | 6.8±2.95 |
|  | Ranibizumab-monthly- 0.5 mg | Laser |  | 7.3±3.22 |
| Payne JF | Ranibizumab-monthly-0.3 mg | Ranibizumab- quarterly- 0.3 mg | 24.70 | 18.90 |
|  | Ranibizumab-monthly-0.3 mg | Laser +Ranibizumab- quarterly- 0.3 mg |  | 17.50 |
| Lang GE | Laser +Ranibizumab- monthly- 0.5 mg | Laser | 5.0±2.1 | 5.2±2.3 |

Note: the treatment method recorded in the table is only the initial treatment plan, and the doctor is allowed to adjust the follow-up treatment plan according to the patient's condition.

**Appendix 3 Summary of the included randomized controlled trials' characteristics related to study quality and the assessment of risk of bias based on these characteristics using the Cochrane Collaboration's tool for assessing risk of bias**

| **author** | **Tool for allocation  Concealment** | **Dropout  rate* %** | **Reason for  dropping out** | **random sequence generation** | **allocation concealment** | **blinding of participants and personnel** | **blinding of outcome assessment** | **incomplete outcome data** | **selective reporting** | **other sources of bias** |
| --- | --- | --- | --- | --- | --- | --- | --- | --- | --- | --- |
| Bressler SB | unclear | 17 | unclear | L | H | L | H | H | H | L |
| Figueira J | unclear | 11.5 | unclear | L | H | L | H | L | L | H^*^ |
| Gross JG | unclear | 40 | Yes | L | H | L | H | L | H | L |
| Sun JK | unclear | 20 | unclear | L | H | L | H | H | H | L |
| Gross JG | unclear | 13 | unclear | L | H | L | H | H | H | L |
| Gross JG | unclear | 10 | unclear | L | H | L | H | H | H | L |
| Susan B. | unclear | 13 | Yes | L | H | L | H | L | H | L |
| Beaulieu WT | unclear | 15 | Yes | L | H | L | H | L | H | L |
| Susan B. | unclear | 8 | Yes | L | H | L | H | H | H | L |
|  |  | 12 |  |  |  |  |  |  |  |  |
| Sivaprasad S | minimisation；concealed before allocation；stratified by site | 4 | Yes | L | L | L | L | L | L | L |
| Wykoff CC NMZB | unclear | 7 | unclear | L | H | L | H | H | H | L |
| Scott IU | permuted block;stratified | 17 | unclear | L | L | L | L | L | L | L |
| Mitchell P | unclear | 12.5 | unclear | L | H | L | L | L | L | L |
|  |  | 12.2 |  |  |  |  |  |  |  |  |
| Payne JF | unclear | 21 | Yes | L | H | L | H | H | H | L |
|  |  |  |  |  |  |  |  |  |  |  |
| Lang GE | unclear | 0 | N/A | L | H | L | L | L | L | H^**^ |

* Eyes in either arm could receive ranibizumab to treat DME at investigator discretion at any time during study participation in this study..

** Premature termination of the trial.

**Appendix 4 Total number of complications**

|  | **PRP** | **Ranibizumab-monthly-0.5 mg** | **PRP + Ranibizumab-quarterly-0.5 mg** | **Aflibercept-quarterly-2 mg** |
| --- | --- | --- | --- | --- |
| **Retinal detachment** | 30/20/2^[11/12/21]^ | 12/11^[11/12]^ | 0^[21]^ | - |
| **Vitreous hemorrhage** | 93/69/9^[11/12/21]^ | 91/52^[11/12]^ | 11^[21]^ | - |
| **Vitrectomy** | 39/30/5^[11/12/21]^ | 21/8^[11/12]^ | 1^[21]^ | - |
| **Nonfatal myocardial infarction** | 2/3/3^[13/14/22]^ | 3/7^[13/14]^ | - | 3^[22]^ |
| **Nonfatal stroke** | 4/4/0^[13/14/22]^ | 2/6^[13/14]^ | - | 3^[22]^ |
| **Death from vascular or unknown cause** | 1/2/1^[13/14/22]^ | 4/6^[13/14]^ | - | 2^[22]^ |

Note: The complications in this form only selected the data recorded in three or more studies at the same time.

**Appendix 5 Rate of complications (%)**

|  | **PRP** | **Ranibizumab-monthly-0.5 mg** | **PRP + Ranibizumab-quarterly-0.5 mg** | **Aflibercept-quarterly-2 mg** |
| --- | --- | --- | --- | --- |
| **Retinal detachment** | 14.8/9.9/4.3^[11/12/21]^ | 6.3/5.8^[11/12]^ | 0^[21]^ | - |
| **Vitreous hemorrhage** | 45.8/34.0/19.5^[11/12/21]^ | 47.6/27.2^[11/12]^ | 26.8^[21]^ | - |
| **Vitrectomy** | 19.2/14.8/10.9^[11/12/21]^ | 11.0/4.2^[11/12]^ | 2.4^[21]^ | - |
| **Nonfatal myocardial infarction** | 1.0/2.6/2.6^[13/14/22]^ | 1.6/6.9^[13/14]^ | - | 2.6^[22]^ |
| **Nonfatal stroke** | 2.0/3.5/0^[13/14/22]^ | 1.0/5.9^[13/14]^ | - | 2.6^[22]^ |
| **Death from vascular or unknown cause** | 0.5/1.7/0.9^[13/14/22]^ | 2.1/5.9^[13/14]^ | - | 1.7^[22]^ |

Note: The complications in this form only selected the data recorded in three or more studies at the same time.

**Appendix 6 Average complication rate (%)**

|  | **PRP** | **Ranibizumab-monthly-0.5 mg** |
| --- | --- | --- |
| **Retinal detachment** | 9.7±4.3^[11/12/21]^ | 6.1±0.3^[11/12]^ |
| **Vitreous hemorrhage** | 33.1±10.8^[11/12/21]^ | 37.4±10.2^11/12]^ |
| **Vitrectomy** | 15.0±3.4^[11/12/21]^ | 7.6±3.4^[11/12]^ |
| **Nonfatal myocardial infarction** | 2.1±0.8^[13/14/22]^ | 4.3±2.7^[13/14]^ |
| **Nonfatal stroke** | 2.8±0.8^[13/14]^ | 3.5±2.5^[13/14]^ |
| **Death from vascular or unknown cause** | 1.0±0.5^[13/14/22]^ | 4.0±1.9^[13/14]^ |
| **Total** | 11.1±12.4 | 10.6±13.0 |

**Appendix 7 Forest plots for comparisons of vision score change.**

**
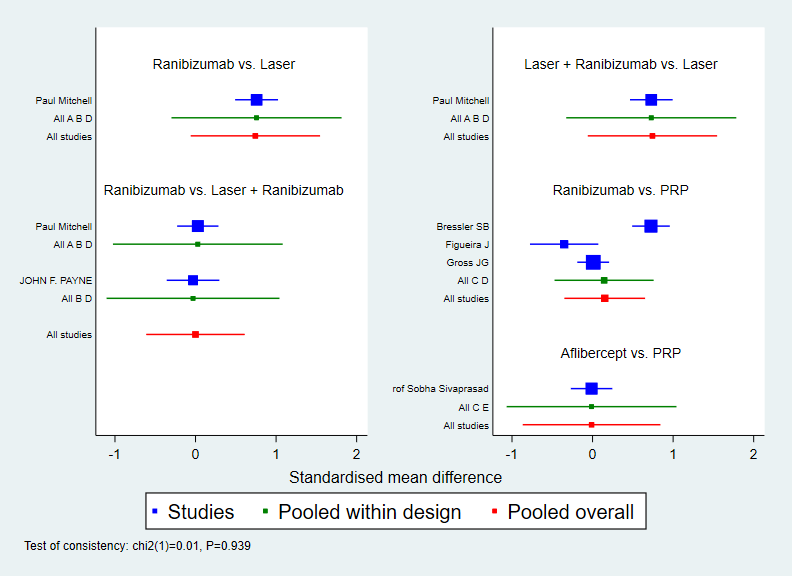
**

Note: *P* value is the result of the inconsistency test. Chi^2^ =  chi square statistic.

**Appendix 8 Contribution map of vision score change.**

**
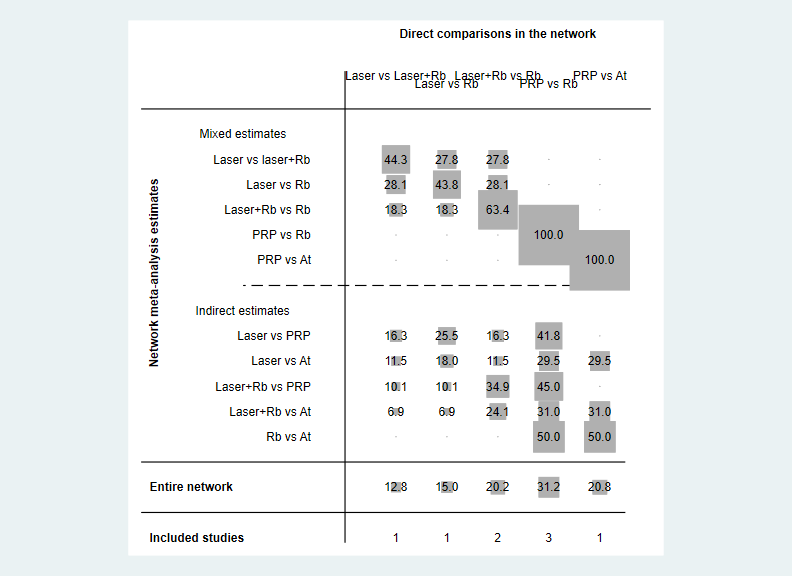
**

Abbreviation: Ranibizumab = Rb; Aflibercept = At;

**Appendix 9 SUCRA value of vision score change.**

**
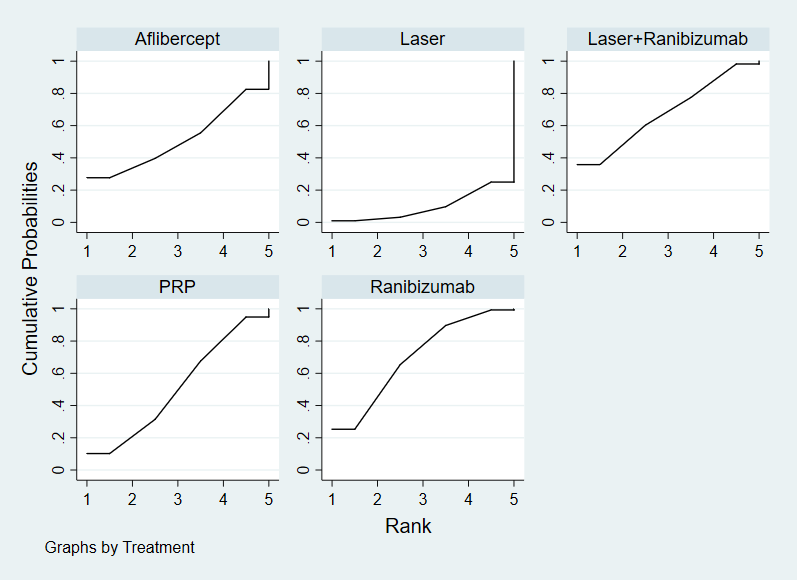
**

The area under the curve is directly proportional to the possibility of the best intervention.

**Appendix 10 Funnel of vision score change.**

**
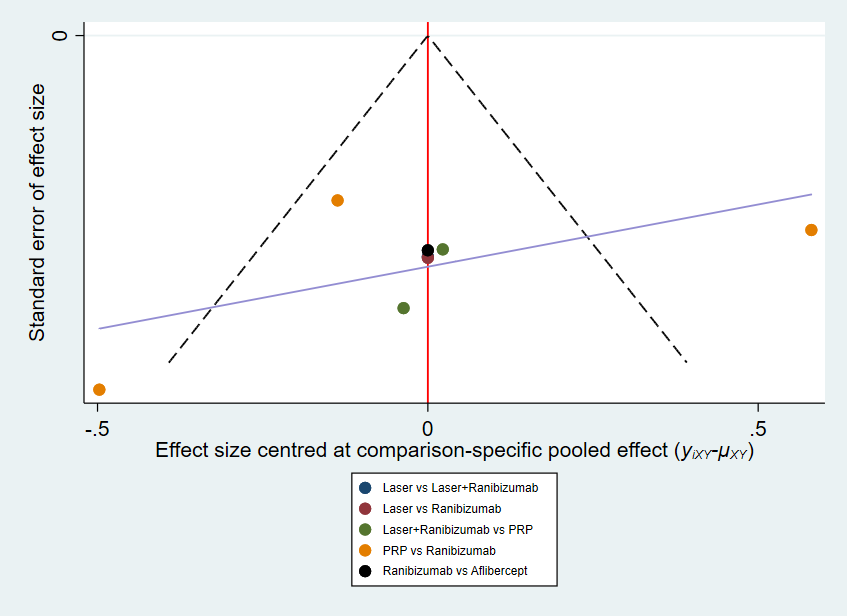
**

**Appendix 11 Begg’s test of vision score change.**

**
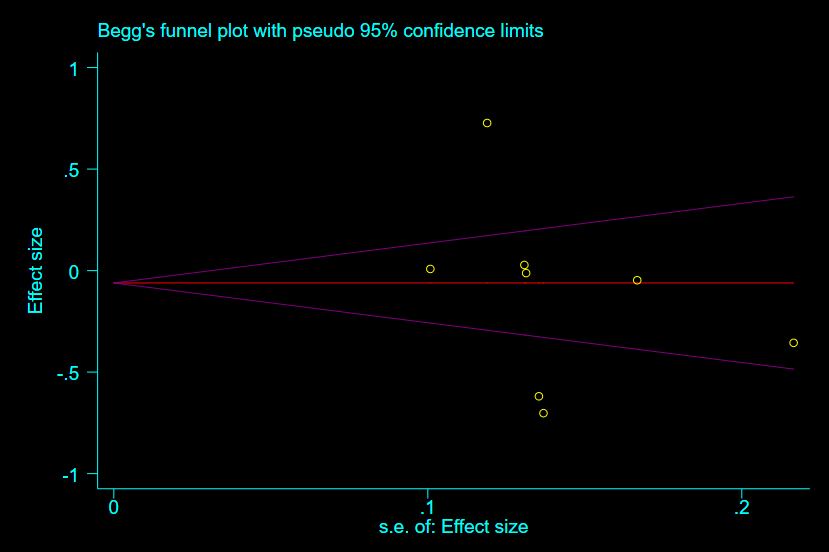
**

**Appendix 12 Egger’s test of vision score change.**

| Std_Eff | Coef | Std_Err. | t | P>\|t\| | [95% Conf | Interval] |
| --- | --- | --- | --- | --- | --- | --- |
| slope | 0.6827049 | 0.8584568 | 0.80 | 0.457 | -1.417863 | 2.783273 |
| bias | -5.692275 | 6.44507 | -0.88 | 0.411 | -21.46279 | 10.07824 |

Abbreviation: Std_Eff  =  standard effect, Coef  =  coefficient, Std_Err.  =  standard error.

**Appendix 13 Network for subgroup analysis of visual score changes.**

**
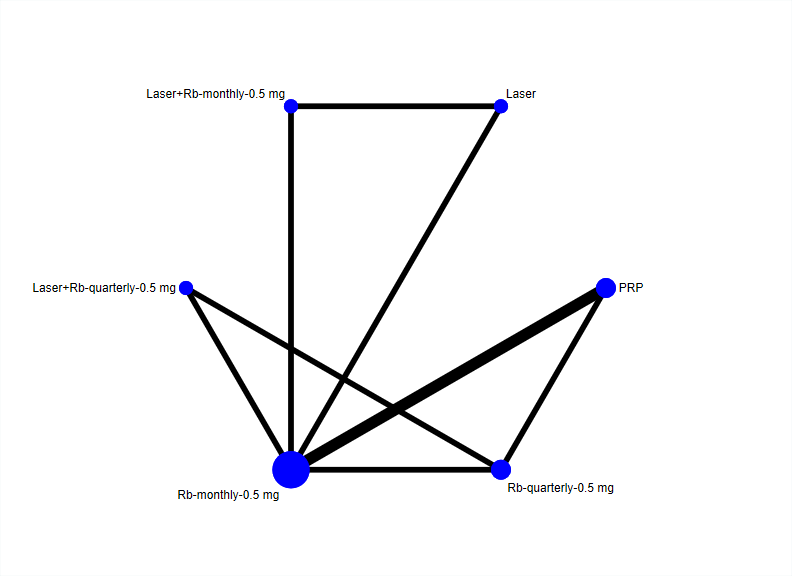
**

Abbreviation: Ranibizumab = Rb;

Seven studies involving 1136 eyes were included. Six interventions were included to form three closed loops. Each circle indicates a treatment node. The size of the nodes is proportional to the number of trials evaluating each treatment. Lines connecting 2 nodes represent direct comparisons between 2 treatments. The thicker the number of lines between nodes, the more research.

**Appendix 14 Forest for subgroup analysis of visual score changes.**

**
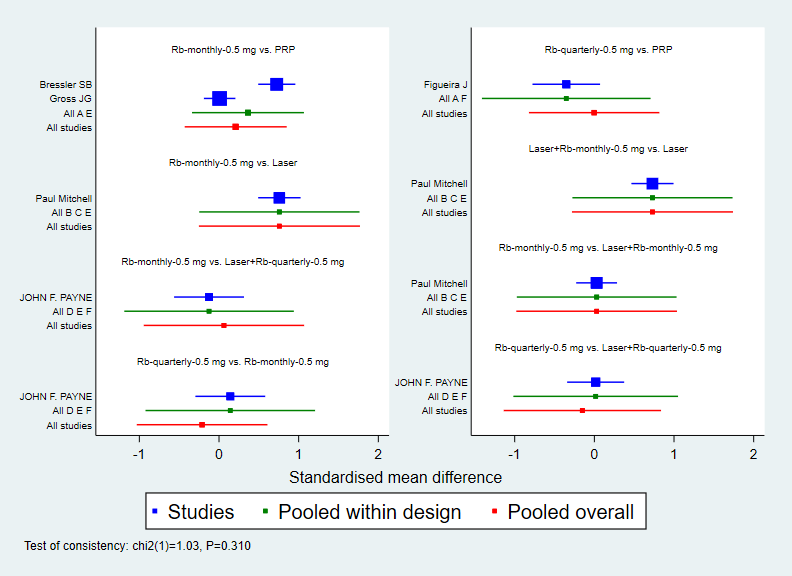
**

Note: *P* value is the result of the inconsistency test.

Abbreviation: Ranibizumab = Rb; Chi^2^ =  chi square statistic.

**Appendix 15 Contribution map for subgroup analysis of vision score change.**

**
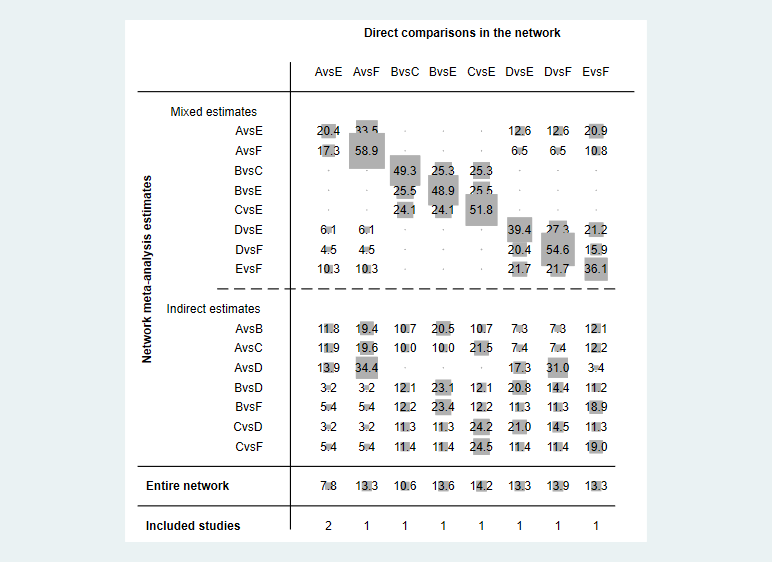
**

Abbreviation: A = PRP; B = Laser; C = Laser + Ranibizumab-monthly-0.5 mg; D = Laser + Ranibizumab-quarterly-0.3 mg; E = Ranibizumab-monthly-0.5 mg; F = Ranibizumab-quarterly-0.3 mg;

**Appendix 16 SUCRA value for subgroup analysis of vision score change.**

**
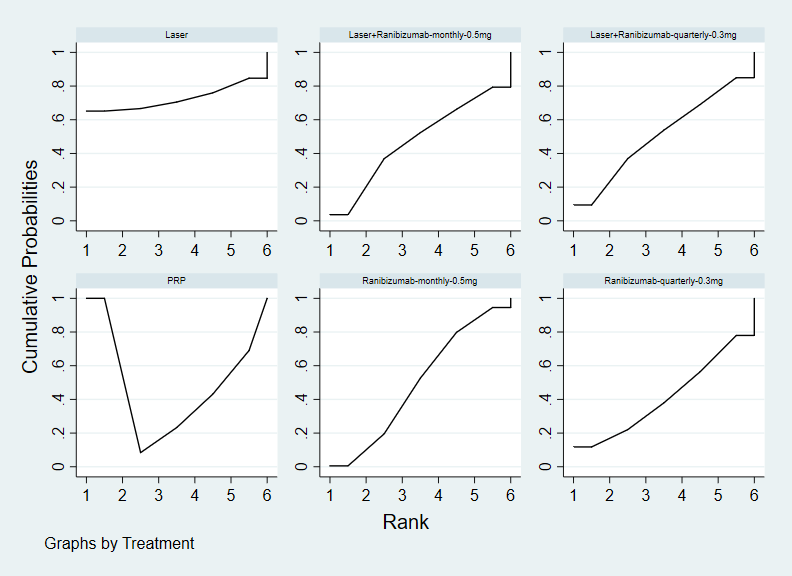
**

The area under the curve is directly proportional to the possibility of the best intervention.

**Appendix 17 Funnel for subgroup analysis of vision score change.**

**
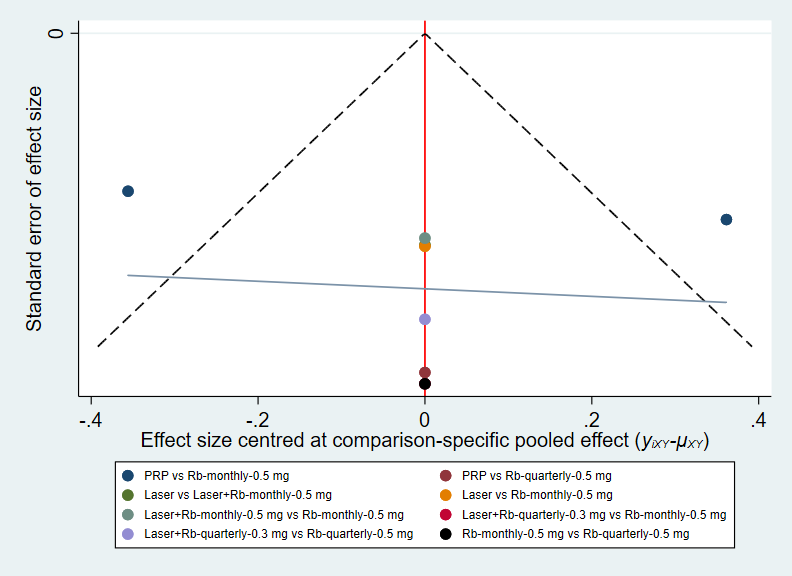
**

**Appendix 18 Network meta-analysis for subgroup analysis of vision score change.**

| Ranibizumab-monthly-0.5mg |  |  |  |  |  |
| --- | --- | --- | --- | --- | --- |
| 0.76 (-0.25,1.76) | Laser |  |  |  |  |
| 0.03 (-0.97,1.03) | -0.73 (-1.74,0.28) | Laser+Ranibizumab-monthly-0.5mg |  |  |  |
| 0.72 (-0.55,1.99) | -0.04 (-1.66,1.58) | 0.69 (-0.93,2.31) | Ranibizumab-quarterly-0.5mg |  |  |
| 0.36 (-0.34,1.07) | -0.39 (-1.62,0.83) | 0.34 (-0.89,1.56) | -0.35 (-1.41,0.71) | PRP |  |
| 0.38 (-0.85,1.60) | -0.38 (-1.97,1.20) | 0.35 (-1.24,1.93) | -0.34 (-1.80,1.12) | 0.01 (-0.99,1.02) | Aflibercept-quarterly-2mg |
| 58.0% | 54.5% | 54.0% | 53.7% | 49.1% | 47.3% |

Treatment reports were sorted according to the degree of vision improvement in subgroups. Comparisons should be read from left to right. The estimate is located at the intersection of the column-defining treatment and the row-defining treatment. Values in parenthesis indicate the 95% CI.

**Appendix 19 Forest plots for comparisons of central retinal thickness.**


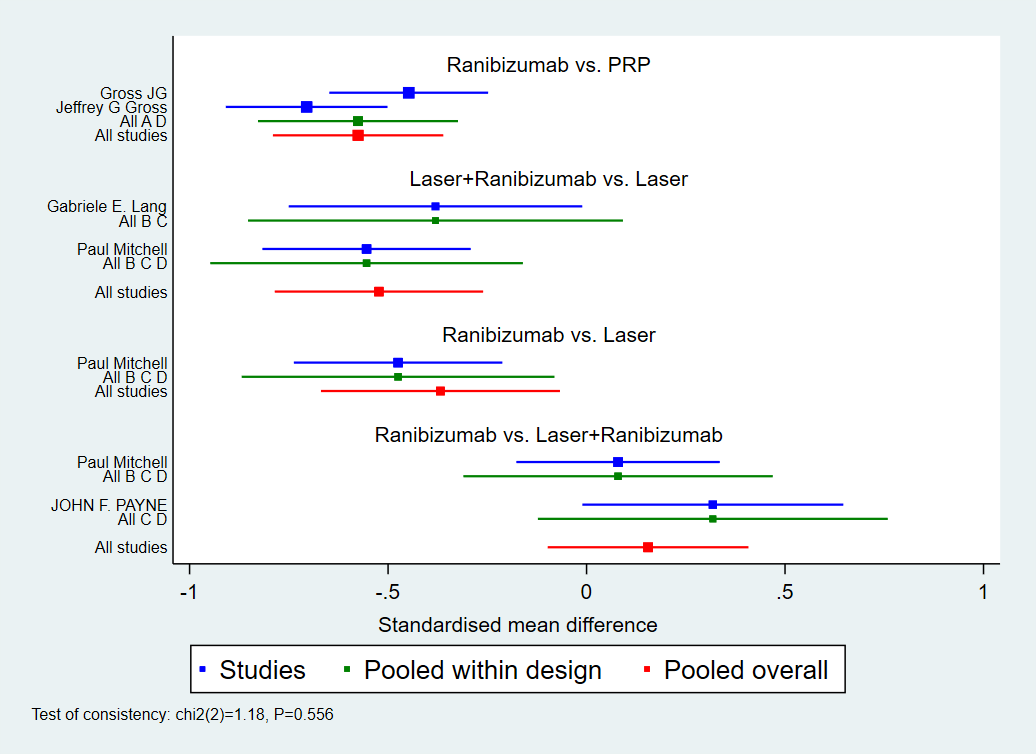


Note: *P* value is the result of the inconsistency test. Chi^2^ =  chi square statistic.

**Appendix 20 Contribution map of central retinal thicknes.**


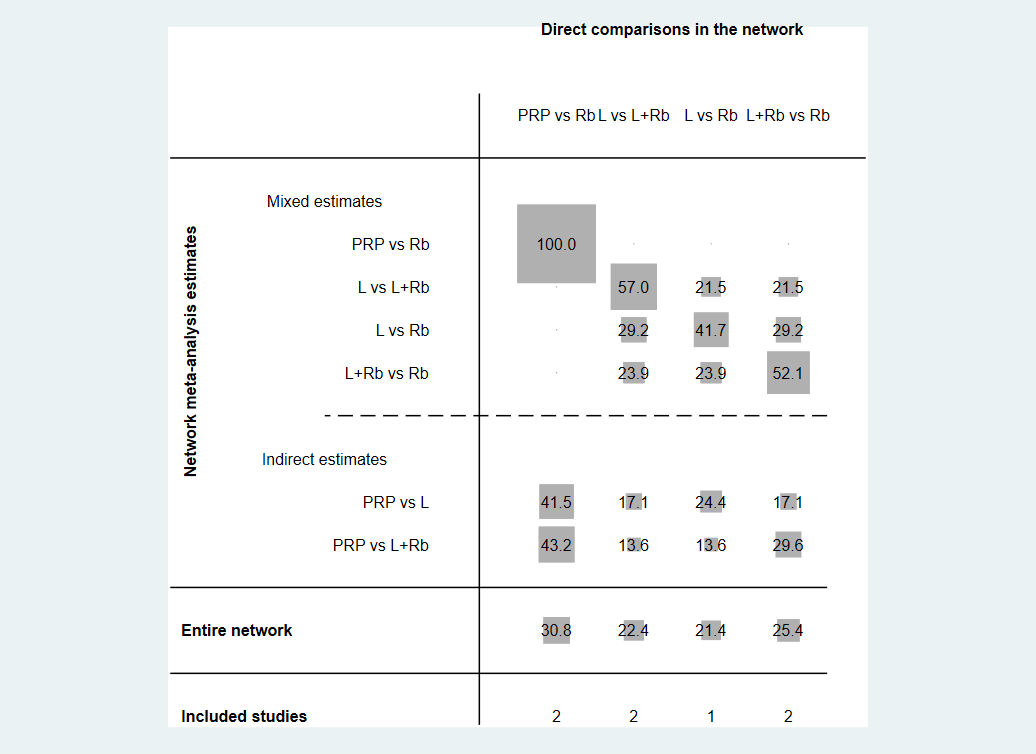


Abbreviation: Ranibizumab = Rb; Laser= L;

**Appendix 21 SUCRA value of central retinal thicknes.**


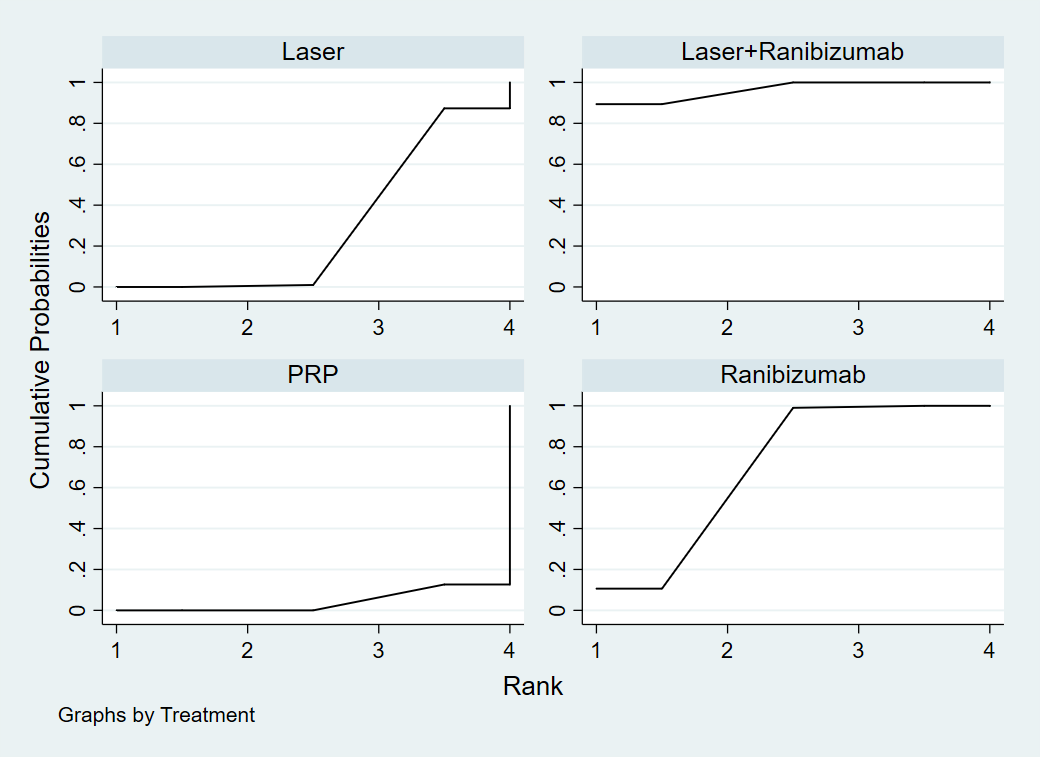


The area under the curve is directly proportional to the possibility of the best intervention.

**Appendix 22 Funnel of central retinal thicknes.**


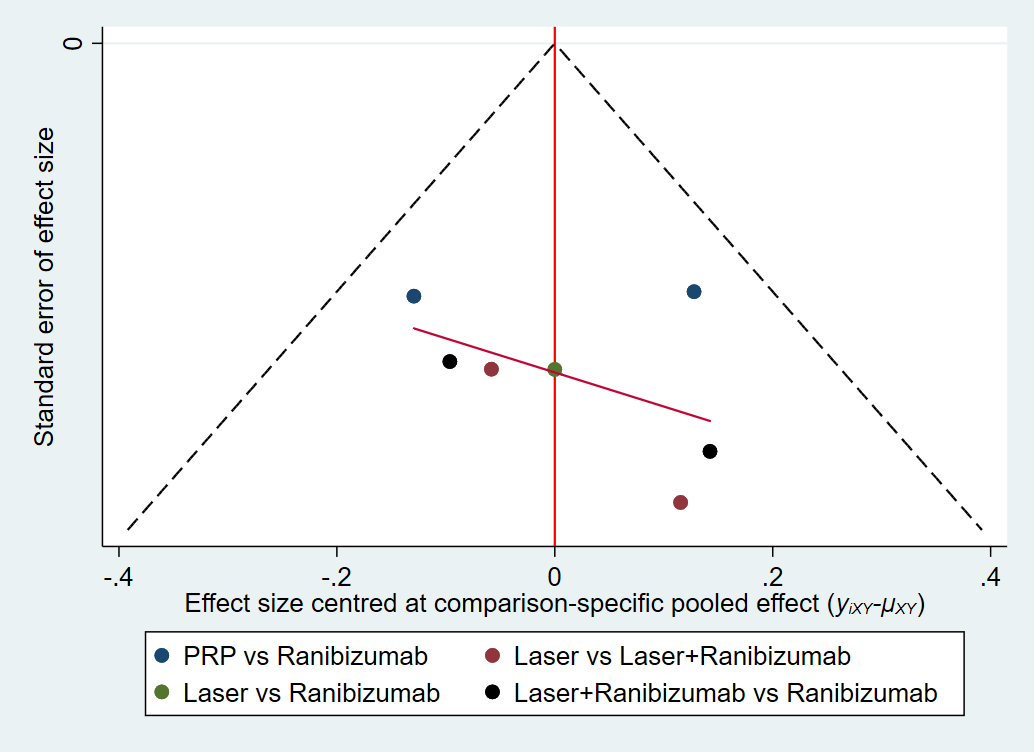


**Appendix 23 Begg’s test of central retinal thicknes.**


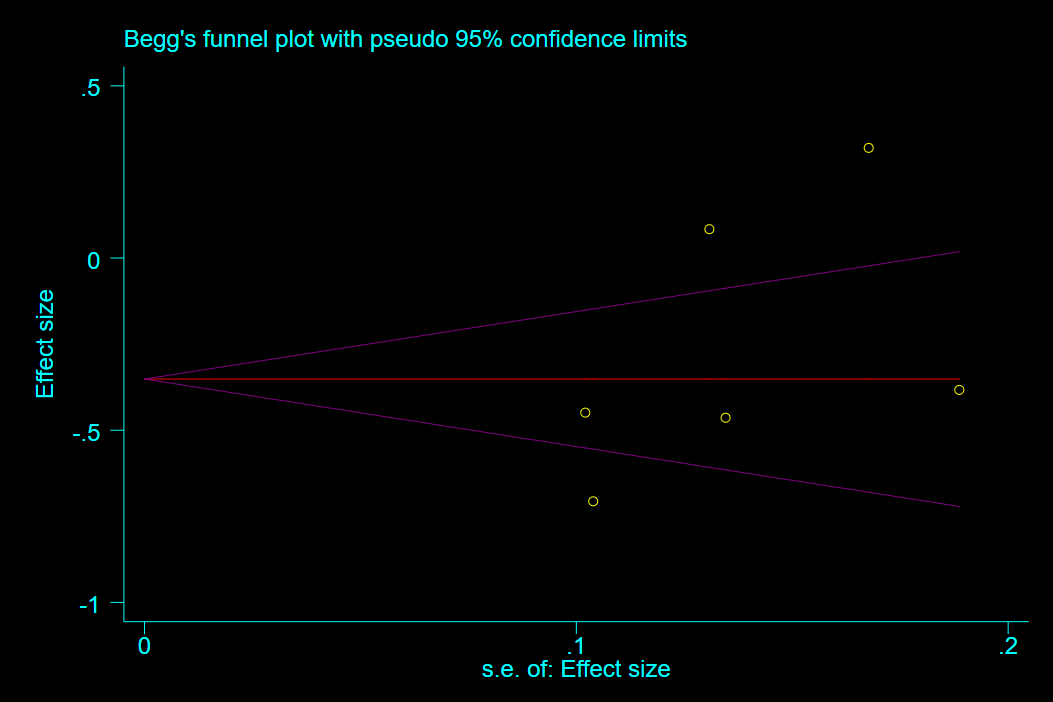


**Appendix 24 Egger’s test of central retinal thicknes.**

| Std_Eff | Coef | Std_Err. | t | P>\|t\| | [95% Conf | Interval] |
| --- | --- | --- | --- | --- | --- | --- |
| slope | -1.234491 | 0.6264913 | -1.97 | 0.120 | -2.973909 | .5049282 |
| bias | 7.061056 | 4.89242 | 1.44 | 0.222 | -6.522481 | 20.64459 |

Abbreviation: Std_Eff  =  standard effect, Coef  =  coefficient, Std_Err.  =  standard error.

**Appendix 25 Network meta-analysis for subgroup analysis of** **central retinal thickness.**


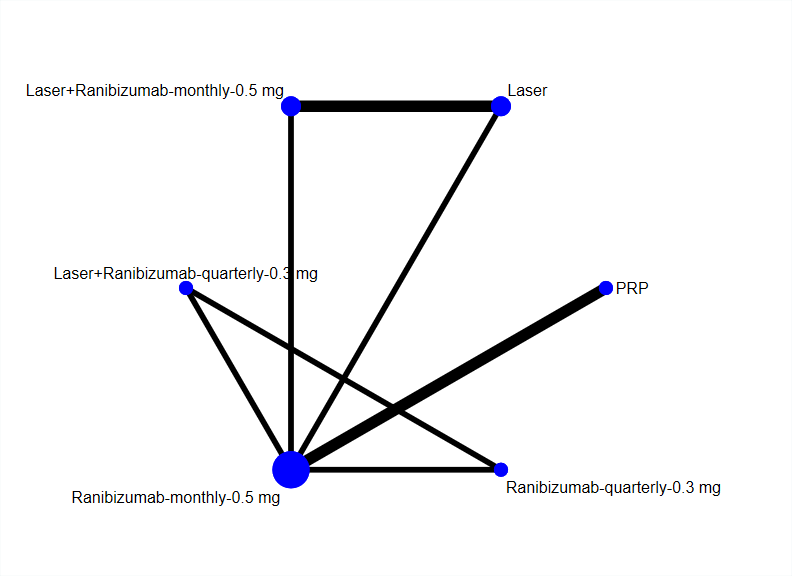


Six studies involving 1411 eyes were included. Six interventions were included to form a closed loop. Each circle indicates a treatment node. The size of the nodes is proportional to the number of trials evaluating each treatment. Lines connecting 2 nodes represent direct comparisons between 2 treatments. The thicker the number of lines between nodes, the more research.

**Appendix 26 Forest for subgroup analysis of central retinal thickness.**


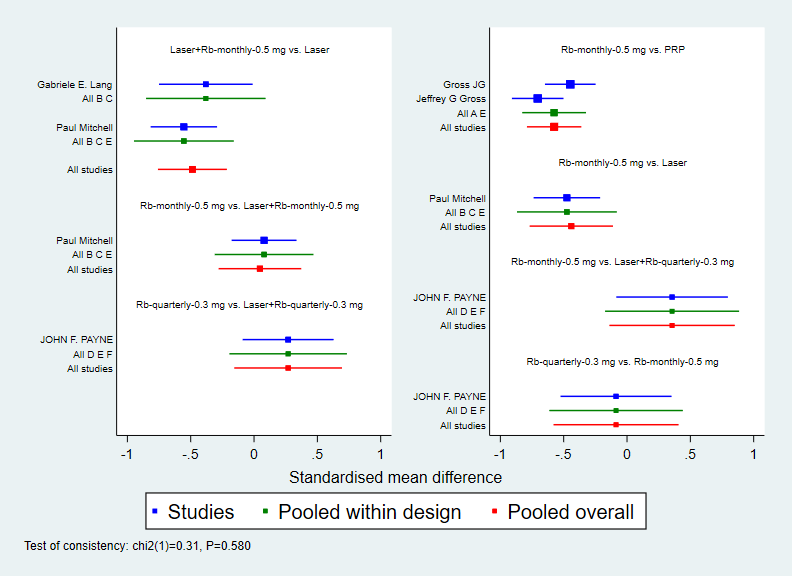


Note: *P* value is the result of the inconsistency test.

Abbreviation: Ranibizumab = Rb; Chi^2^ =  chi square statistic.

**Appendix 27 SUCRA value for subgroup analysis of central retinal thickness.**


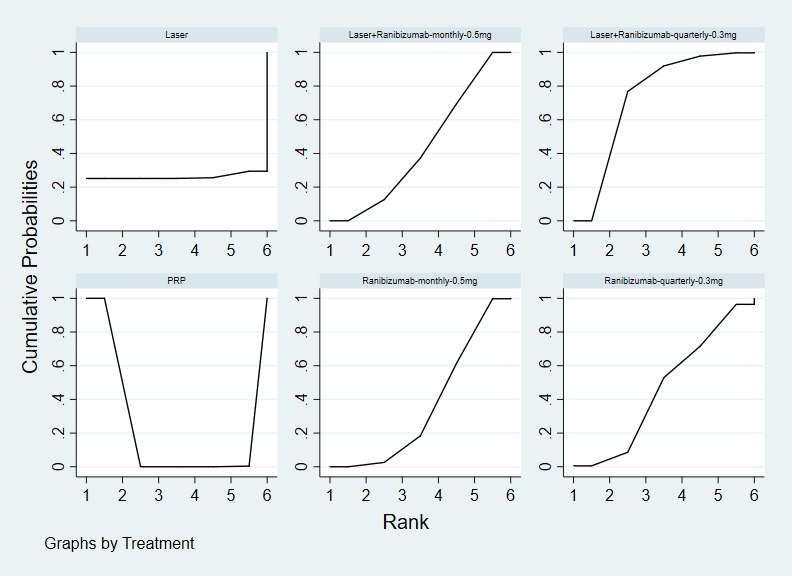


The area under the curve is directly proportional to the possibility of the best intervention.

**Appendix 28 Funnel for subgroup analysis of central retinal thickness.**


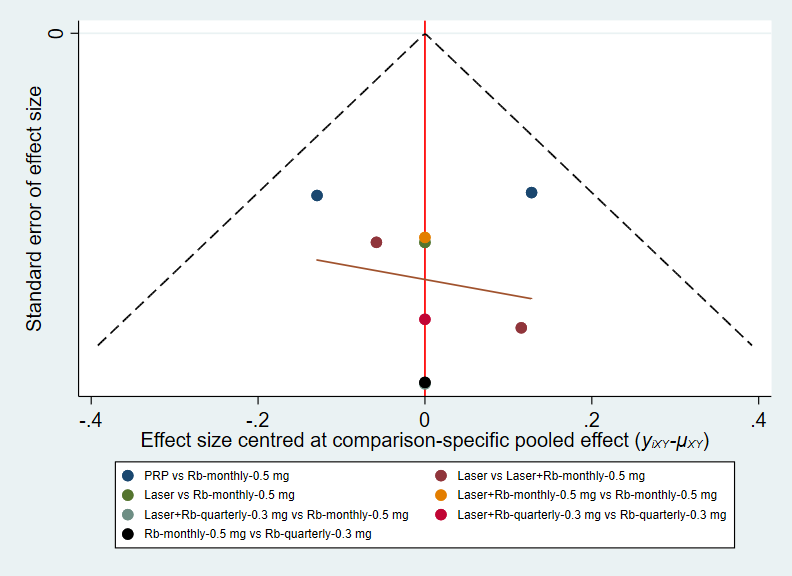


Abbreviation: Ranibizumab = Rb.

**Appendix 29 Network meta-analysis for subgroup analysis of central retinal thickness.**

| Laser+Rb-quarterly-0.3mg |  |  |  |  |  |
| --- | --- | --- | --- | --- | --- |
| -0.27 (-0.69,0.16) | Rb-quarterly-0.3mg |  |  |  |  |
| -0.31 (-0.90,0.28) | -0.04 (-0.63,0.55) | Laser+Rb-monthly-0.5mg |  |  |  |
| -0.36 (-0.85,0.14) | -0.09 (-0.58,0.41) | -0.05 (-0.37,0.28) | Rb-monthly-0.5mg |  |  |
| -0.80 (-1.39,-0.20) | -0.53 (-1.12,0.07) | -0.49 (-0.76,-0.21) | -0.44 (-0.77,-0.11) | Laser |  |
| -0.93 (-1.47,-0.39) | -0.66 (-1.20,-0.12) | -0.62 (-1.01,-0.23) | -0.58 (-0.79,-0.36) | -0.14 (-0.53,0.26) | PRP |
| 72.7% | 45.7% | 44.3% | 36.8% | 26.2% | 20.1% |

Treatment reports were sorted according to the degree of vision improvement in subgroups. Comparisons should be read from left to right. The estimate is located at the intersection of the column-defining treatment and the row-defining treatment. Values in parenthesis indicate the 95% CI.
